# Supplementary material for: In Situ Corn Fiber Conversion for Ethanol Improvement by the Addition of a Novel Lignocellulolytic Enzyme Cocktail
Source: J Fungi (Basel). 2022 Feb 24;8(3):221. doi: 10.3390/jof8030221 (PMC8951183; doi:10.3390/jof8030221)

Supplemental Figure S1 The color change of DDGS with/without lignocellulolytic enzymes addition.

without lignocellulolytic enzymes addition.

without lignocellulolytic enzymes addition.

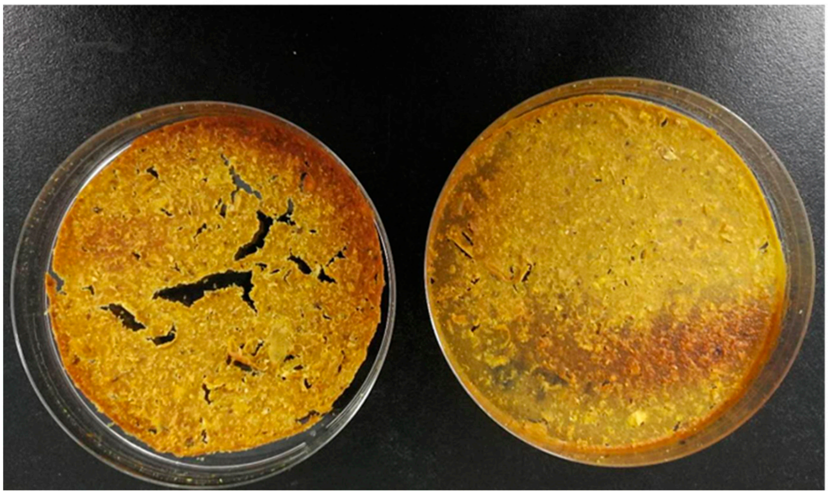

Supplemental Figure S2 The effect of different enzyme loadings on ethanol yield

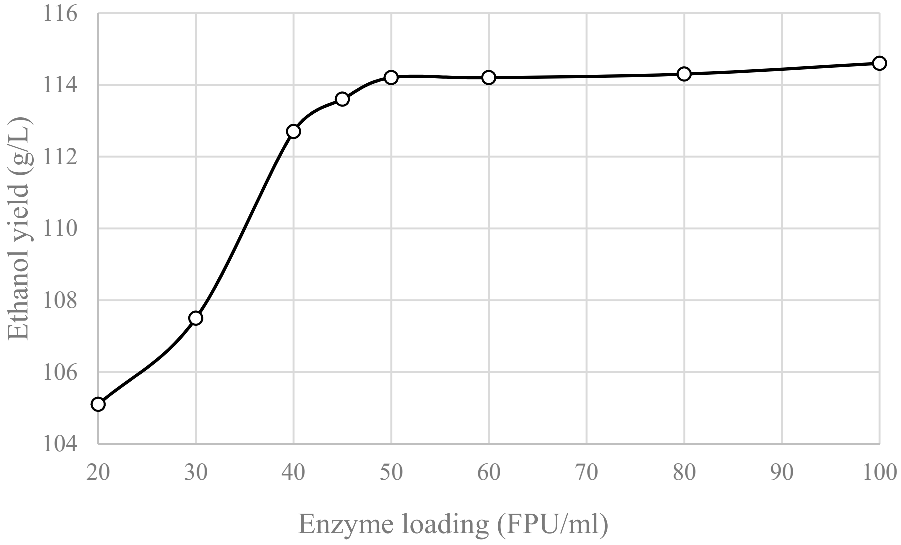

Supplement: Supplementary file 1 [file jof-08-00221-s001.zip › jof-1606809-supplementary.pdf]
